# Adherence to the referral advice after introduction of rectal artesunate for pre-referral treatment of severe malaria at the community level: a noninferiority trial in the Democratic Republic of the Congo

**DOI:** 10.1186/s12936-019-3074-6

**Published:** 2019-12-21

**Authors:** Patrick M. Mvumbi, Jeanine Musau, Ousmane Faye, Hyppolite Situakibanza, Emile Okitolonda

**Affiliations:** 1 School of Public Health, Kinshasa, Democratic Republic of the Congo; 2 Management Science for Health, Integrated Health Project (IHP), Kinshasa, Democratic Republic of the Congo; 3 Department of Tropical Medicine, University of Kinshasa, Kinshasa, Democratic Republic of the Congo

**Keywords:** Rectal artesunate, Prereferral treatment, Severe malaria, Community level, Community health workers, DRC

## Abstract

**Background:**

The Democratic Republic of the Congo adopted the strategy of using, at the community level, a dose of rectal artesunate as a pre-referral treatment for severe malaria amongst children under 5 years who could not quickly reach a health care facility and take oral medication. However, the adherence to referral advice after the integration of this strategy and the acceptability of the strategy were unknown.

**Methods:**

To assess adherence by the mothers/caretakers of children under 5 years to referral advice provided by the community health workers after pre-referral treatment of severe malaria with rectal artesunate, the authors conducted a noninferiority community trial with a pre- and post-intervention design in 63 (pre-intervention) and 51 (post-intervention) community care sites in 4 provinces (Kasaï-Oriental, Kasaï-Central, Lomami, Lualaba) from August 2014 through June 2016. The pre- and post-intervention surveys targets 387 mothers of children under 5 years and 63 community health workers and 346 mothers and 41 community health workers, respectively. A 15% margin was considered for noninferiority analyses due to the expected decrease in adherence to referral advice after the introduction of the new intervention.

**Results:**

The mothers acknowledged that the rectal route was often used (60.7%), and medicines given rectally were considered more effective (63.6%) and easy to administer (69.7%). The acceptability of pre-referral rectal artesunate was relatively high: 79.4% (95% CI 75.4–83.3) among mothers, 90.3% (95% CI 82.3–96.8) among community health workers, and 97.8% (95% CI 93.3–100) among nurses. Adherence to referral advice at post-intervention [84.3% (95% CI 80.6–88.1)] was non-inferior to pre-intervention adherence [94.1% (95% CI 91.7–96.4)].

**Conclusions:**

The integration of pre-referral rectal artesunate for severe malaria into the community care site in the DR Congo is feasible and acceptable. It positively affected adherence to referral advice. However, more health education is needed for parents of children under 5 years and community health workers.

## Background

Globally, an estimated 435,000 people died of malaria in 2017. Approximately 61% of the victims were children under 5 years (CU5), and 93% of those malaria deaths occurred in the World Health Organization (WHO) African Region. Among the 17 countries that had nearly 80% of the malaria deaths globally in 2017, Nigeria accounted for the highest proportion of deaths (19%), followed by the Democratic Republic of Congo (DRC) (11%) [1]. Approximately six to eight deaths out of 10 occur in the community before that patient reaches a health facility or within 30 min after arrival at the health facility [2].

Severe malaria progresses in children very quickly, and once a patient can no longer be given oral medication, survival depends upon quickly being taken to the hospital, where parenteral treatment can be given. Parenteral anti-malarial treatment is sometimes available at the primary health care level. However, access to parenteral treatment is quite difficult, and the delay can have a negative impact on the progress of the disease, even leading to death. Because the disease progresses rapidly, the risk of death from severe malaria is greatest in the first 24 h of the onset of symptoms [3], yet in most malaria endemic countries, the transit time between referral and arrival at health facilities that can administer intravenous treatment is usually prolonged, thereby delaying the commencement of appropriate anti-malarial treatment.

Therefore, the 2010 WHO guidelines for the treatment of malaria recommend the use of an emergency treatment for patients with suspected severe malaria before their transfer to a health facility. The WHO-recommended pre-referral treatment options include intramuscular artesunate, artemether or quinine, or rectal artesunate (RA) [4].

Evidence from recent studies demonstrates that in situations where IV administration is not possible and intramuscular injection is impractical (and the patient cannot be treated orally), the use of a single dose of RA as pre-referral treatment reduces the risk of death or permanent disability in young children [5]. The use of this intervention in endemic countries remains low, pending evidence about its efficacy, effectiveness and cost-effectiveness.

In line with the WHO recommendation, the DRC adopted the strategy in 2012 but delayed its implementation because its acceptability and feasibility were still unknown. In its effort to reduce the morbidity and mortality of malaria, the DRC in 2005 launched the community-based management of malaria integrated with pneumonia and diarrhoea at community care sites (CCSs) within the framework of the Integrated Management of Childhood Illness (IMCI).

The cornerstone of this strategy is the early detection and prompt treatment of clinical cases, and CHWs are given the opportunity to diagnose and treat malaria. This requires that effective and appropriate treatment with first-line oral artemisinin-based combination therapy (ACT) and guidance on referral criteria are provided at the community level through trained CHWs. Community-based management of malaria allows the coverage of health services for malaria to extend beyond the reach of health facilities. Diagnosis with a rapid diagnostic test (RDT) prior to the treatment of malaria-positive patients with ACT is currently the recommended practice. Severe cases should be referred to an appropriate facility.

CCSs are well-defined geographic areas covering one or more villages that are far from a health facility (more than 5 km) and that receive health care services provided by two trained CHWs. The CHWs are trained in IMCI and are selected and appointed by the communities. They provide the following services under the supervision of the chief nurse of the health facility (primary point of care): community management of childhood diseases (malaria, diarrhea, pneumonia and malnutrition), including the referral of cases with danger signs; community outreach activities; and community-based surveillance of diseases. Some studies have shown that in some countries, it was feasible and acceptable to use rectal artesunate at the community level as a pre-referral treatment for severe malaria [6–9]. In contrast, in Laos, the rectal route was found unacceptable by the population in general, and health care workers (HCWs) impeded the use of a pre-referral emergency malaria treatment [10].

Although the feasibility and acceptability of the strategy has been demonstrated, its effect on adherence to referral advice has not been thoroughly assessed. Vermeersch et al. noted the need to address the mother’s judgement of the severity of malaria to prevent overuse of the RA as a pre-referral treatment [9]. Moreover, Kaona et al. outlined that gaining insights into the mother’s capacity to distinguish uncomplicated malaria from severe symptoms of the disease is key to successfully implementing pre-referral treatment at the community level [6]. On the other hand, adherence to referral advice is a cornerstone of the success of the strategy. Indeed, concerns have been raised regarding whether the risks of routine use of pre-referral RA at the community level include not only failure to adhere to medical advice, as the child will recover rapidly [8, 9, 11], but also the increasing possibility of artemisinin-based drug resistance [8, 12, 13]. However, there is limited information on adherence to referral advice following the administration of RA for pre-referral treatment of severe malaria [11]. Overall, most studies assessed adherence to referrals for any serious illness conditions from either the community level to health facilities [14] or from primary health facilities to hospitals [15–19].

The primary objective of this study was to assess the adherence of the mothers/caretakers of CU5 to referral advice after pre-referral treatment of severe malaria with RA at the community level. The secondary objectives were to determine the acceptability of the strategy and the knowledge of CHWs regarding danger signs of malaria to better inform scaling up. The main hypothesis was that the integration of pre-referral RA at the community level will not negatively affect the uptake of referrals of CU5 with symptoms of severe malaria, i.e., adherence to the referral advice after the introduction of the “new intervention” (a pre-referral RA for CU5 with danger signs of malaria) will be noninferior by more than a 15% margin compared to adherence to “reference intervention” (urgently referring CU5 with malaria danger signs without providing any treatment).

The introduction of RA as a pre-referral treatment for severe malaria might pose the risk that the child will not be taken to a health facility because he or she will appear to get better rapidly after the treatment; however, such non-adherence runs the risk of death from resurgence and the development of drug resistance due to incomplete removal of parasites. However, pre-referral RA is more acceptable because it prevents the danger of death and disability, taking into account that every hour without treatment for severe malaria increases the risk of death [3]. This study does not add to the knowledge of the efficacy or cost-effectiveness, but could add some evidence of the effectiveness of RA.

## Methods

### Study design

A noninferiority community trial with a pre- and post-intervention design was conducted from August 2014 through June 2016 to determine whether the integration of RA for the pre-referral treatment of severe malaria (hereby referred to as the new intervention) within the routine IMCI (referred to as the reference intervention) will not be worse than the reference intervention regarding adherence to referral advice. The margin of noninferiority selected was 15%, i.e., adherence to referral advice at post-intervention will not be more inferior than 15% compared to the pre-intervention adherence.

It was assumed that the new intervention would decrease adherence to referral advice because the pre-referral RA, when given promptly in the community, is proven to act rapidly on the danger signs [5], and rapid recovery of the CU5 may lead mothers/caretakers to perceive that no further treatment is necessary [8]. To be successful, this strategy must be followed by a quick referral to a health center or hospital with adequate facilities for anti-malarial injections and supportive care. However, the new intervention, with respect to the reference intervention, is of interest because it has some other advantages, such as reducing death and permanent disability due to severe malaria among children under five (CU5) [5].

### Study sites and participants

The study was conducted at CCSs in rural areas (each with two CHWs) located far from health facilities (distances > 5 km and/or separation by natural barriers). The study took place in 63 CCSs (pre-intervention) and 51 CCSs (post-intervention) located in seven health districts, namely, Kanda-Kanda (in the province of West Kasai); Bibanga (Lomami); Bilomba, Lubondaie and Dibaya (Kasai); Fungurume and Lualaba (Lualaba). At post-intervention, the two latter health districts (Fungurume and Lualaba) were excluded from the sampling frame due to budgetary constraints. All health districts were located in high malaria transmission areas, and they were supported for malaria prevention and management interventions by the Integrated Health Project in DRC (IHP) funded by the President’s Malaria Initiative (PMI) and implemented by Management Science for Health (MSH). The CCSs were randomly selected from among villages located in remote areas of the health districts and where there were some trained CHWs providing IMCI care to CU5. At each CCS, one CHW was selected to participate in the study.

The primary concern with the implementation of RA at the community level is that referral completion rates will decrease, as caregivers might not realize the necessity of completing referrals because the children might improve clinically and/or because they have already received a medication from the CHW; given this operational concern, a noninferiority analysis was the most appropriate method for sample size and power calculations. This analysis permits researchers to assess whether a given outcome level (for example, post-RA rollout referral completion rates) is “noninferior” to a designated outcome level (e.g., pre-RA rollout referral completion rates) within a certain margin of error.

Assuming a 50% referral completion rate to be conservative and a 15% noninferiority limit (e.g., post-RA roll-out referral completion rates are no more than 15% “worse” (lower) than pre-RA roll-out completion rates), 80% power, a Type I error of 0.05, intraclass correlation of 0.05 (from clustering of the referral outcome at the CHW level), sample sizes of 64 CHWs and 384 caregivers were needed based on estimates that 5% of the children seen per month have danger signs (as recorded in CHW registers) and that 45% of participants will complete the follow-up referral.

The eligibility criteria for CU5 to be included in the study were as follows: children aged from 12 to 59 months; with fever (or history of fever within 2 days) plus at least one of the following danger signs for malaria (per the standardized national IMCI guidelines): (i) convulsions; (ii) inability to drink, eat or suck; (iii) vomiting all liquids and solids; (iv) altered consciousness/coma; (v) lethargy.

At pre-intervention, 420 CU5 who sought consultations from September 2014 through April 2014 at 63 CCSs and who were given referral advice because of the presence of fever (or a history of fever in the last 2 days) and at least one danger sign of malaria were randomly selected and included in the study. A total of 384 mothers/caretakers (91.4%) of CU5 were successfully located and interviewed. After three unsuccessful visits to the household during the study period, the remaining 36 mothers/caretakers of CU5 were excluded. At post-intervention, 346 mothers/caretakers of CU5 who consulted at the CCSs were interviewed. In addition, 63 CHWs and 41 CHWs were included in during the pre- and post-intervention periods, respectively.

### Description of the intervention

This was a pilot implementation conducted in rural settings in which care-seeking at health facilities was low and was substantially delayed due to poor access. The principal intervention of interest was the addition to the IMCI package of a single pre-referral dose of RA administered by a CHW to a CU5 with suspected severe malaria and referral advice given to mothers/caretakers. As per the standardized national IMCI guidelines, all children aged 2 months to 59 months who presented at the CCS with fever (or a history of fever in the past 2 days) and at least one danger sign of malaria received one single dose of artesunate suppository corresponding to their age as a pre-referral treatment without being tested for malaria by an RDT. In addition, the CHWs advised the children’s mothers/caretakers to immediately go to the nearest health facility for comprehensive treatment for severe malaria. There was no child in the sample who experienced a side effect after receiving the pre-referral RA for severe malaria or who died.

The CCSs selected for this study have been functioning for a few years and have managed uncomplicated malaria, diarrhoea and pneumonia. With the integration of severe malaria treatment (artesunate suppositories as a pre-referral treatment for CU5) into the IMCI package, the CHWs were trained on this new topic. This training also covered how to correctly fill out various health forms, such as case record forms, to ensure the quality of the data that were collected.

The intervention was implemented from May 2015 through June 2016. Specifically, it consisted of the following activities: (i) refresher trainings of CHWs and chief nurses (those in charge of overseeing CHWs) on the correct identification of the five danger signs of malaria and the correct administration of the rectal artesunate; (ii) pre-referral treatment of eligible CU5 with rectal artesunate; (iii) provision of referral advice to the mothers/caretakers of CU5 to proceed to the nearest health facility for comprehensive severe malaria treatment; (iv) post-referral home visits to check for adherence to referral advice and the status of the referred CU5; (v) following the routine health system’s supply chain management, regular supply of selected CCSs with artesunate suppositories from the chief nurse at the nearest health facility; (vi) regular supervision visits of the CCS conducted by the chief nurses and by the study team.

During the training sessions, in addition to the themes developed in the context of standardized national IMCI guidelines, the focus was on identifying the danger signs and management of severe malaria with pre-referral artesunate suppository in CU5 and referral counselling. The CHW training concluded with a post-training test of their knowledge and skills. CHWs who passed this test were certified to include the prereferral RA in their tasks. Those who did not pass were given the opportunity to obtain additional training and retest. Certification was required to manage severe malaria with RA and participate as a CHW in this intervention study.

As severe malaria is a relatively rare condition, it was important to understand how well-certified CHWs could use and retain their competencies. Thus, monthly evaluations and post-training sessions were provided to the selected CHWs by trained health providers, such as nurses or doctors in targeted health zones. In addition, on a quarterly basis, the trained health providers, along with the study team (supervisors), went to the targeted CCSs to review the case record forms that CHWs complete for each suspected case of severe malaria. This group specifically looked for errors related to case identification, doses given, expulsion of the suppository, referral advice provided, and referral slips given to caregivers. These supervisors also tested the CHWs’ knowledge regarding the identification of cases through scenarios involving hypothetical cases of CU5 presenting with various symptoms, administration of RA treatment, referral of patients to the hospital, and follow-up after patients returned home.

### Data collection

Data collection was carried out in two phases: pre-intervention and post-intervention. At pre-intervention, data on perceptions of severe malaria, history of treatment via the rectal route and acceptability of the pre-referral treatment were collected through a mixed approach (qualitative and quantitative). The results presented in this article will be limited to the quantitative approach, which consisted of conducting interviews of the mothers/caretakers of CU5, CHWs and chief nurses with a structured questionnaire.

During the two study periods, data on adherence to referral advice were assessed based on self-reports by the mothers/caretakers of CU5 that they went to the nearest health facility for a comprehensive treatment according to the referral advice they received. In addition, at post-intervention, data on adherence to referrals were double-checked with follow-up interviews (performed by CHWs 7 days after the child received RA) and interviews conducted by the study team at the end of the study period.

### Data analysis

Data collected through the quantitative approach were recorded using Epidata software ver 3.1 and analysed with SPSS version 20. Proportions with 95% confidence intervals were calculated for the primary outcome (adherence to the referral advice) and secondary outcomes (acceptability of pre-referral rectal artesunate and knowledge of all five danger signs of malaria per the standardized IMCI classification). Comparisons of proportions between pre-intervention and post-intervention for CHWs who knew all five danger signs of malaria were performed using the paired t-test. In addition, the z test for the comparison of differences between two proportions was used for noninferiority analysis with a 15% margin (Δ).

Bivariate analysis using the Pearson (or Fisher exact) Chi-square test was used to assess the association between key independent variables (sociodemographic characteristics, symptoms of children, time of treatment-seeking at CCS) and the dependent variable (adherence to the referral advice). Multiple logistic regression analysis was performed to identify variables (sociodemographics, presenting symptoms of the CU5 at arrival at CCS) independently associated with adherence to referral advice.

## Results

### Acceptability of the pre-referral RA

The sociodemographic characteristics of the mothers/caretakers and CHWs are shown in Tables 1 and 2. At pre-intervention, 84.7% of the respondents (n = 384) were identified as mothers of CU5, compared to 80.6% (n = 346) at post-intervention. Malaria was recognized by the majority of respondents (98.7%) as a public health problem in the community that seriously affected CU5 and led to death.

**Table 1:** Sociodemographic characteristics of the mothers/caretakers of children under 5 years

	Pre-intervention n (%)	Post-intervention n (%)
Sex		
Female	352 (91)	294 (85)
Male	35 (9)	52 (15)
Total	387 (100)	346 (100)
Age (years)		
Median	28	31
Interquartile range (IQR)	13	13
Relationship to CU5		
Mother	328 (84.7)	279 (80.6)
Father	34 (8.8)	47 (13.6)
Grandmother/aunt/stepmother	15 (3.9)	16 (4.6)
Sister/brother/cousin	9 (2.3)	4 (1.2)
Other	1 (0.3)	–
Total	387 (100)	346 (100)
Educational level		
Primary	171 (44.2)	156 (45.1)
High school	85 (22.0)	77 (22.2)
University	–	3 (0.9)
Without education	131 (33.8)	110 (31.8)
Total	387 (100)	346 (100)
Marital status		
Married/marital union	357 (92.3)	329 (95.1)
Single	13 (3.3)	4 (1.2)
Divorced	4 (1.0)	6 (1.7)
Widower	13 (3.4)	7 (2.0)
Total	387 (100)	346 (100)
Occupation		
Farmer	310 (80.1)	321 (92.8)
Unemployed	38 (9.8)	8 (2.3)
Civil servant/teacher/health care worker	16 (4.2)	12 (3.5)
Merchant	12 (3.1)	4 (1.1)
Homemaker	9 (2.3)	–
Dressmaker	2 (0.5)	1 (0.3)
Total	387 (100)	346 (100)

**Table 2:** Sociodemographic characteristics of the community health care workers

	Pre-intervention n (%)	Post-intervention n (%)
Sex		
Male	59 (93.7)	40 (97.6)
Female	4 (6.3)	1 (2.4)
Total	63 (100)	41 (100)
Age (years)		
Median	41	46
Interquartile range (IQR)	15	19
Educational level		
Primary	2 (3.2)	–
High school	58 (92.0)	39 (95.1)
University	2 (3.2)	2 (4.9)
Without education	1 (1.6)	–
Total	63 (100)	41 (100)
Marital status		
Married/marital union	58 (92.0)	40 (97.6)
Single	3 (4.8)	1 (2.4)
Divorced	1 (1.6)	–
Widower	1 (1.6)	–
Total	63 (100)	41 (100)
Occupation		
Farmer	40 (63.4)	24 (58.5)
Teacher	11 (17.5)	7 (17.1)
Pastor	9 (12.7)	4 (9.8)
Unemployed	1 (1.6)	5 (12.2)
Merchant	1 (1.6)	1 (2.4)
Dressmaker	1 (1.6)	–
Blacksmith	1 (1.6)	–
Total	63 (100)	41 (100)

The interviewed mothers stated that the rectal route was often used (60.7%), and medicines given rectally were considered more effective (63.6%) and easy to administer (69.7%). When asked about prior knowledge of the rectal artesunate, 8.7% of the mothers/caretakers reported that they had already heard about it, mainly through CHWs (75%) or family members (25%). In addition, the interviewees were asked if they could use RA for the pre-referral treatment of severe malaria in their CU5 when it was offered by the CHW. The acceptability of RA was relatively high: 79.4% (95% CI 75.4–83.3) among mothers, 90.3% (95% CI 82.3–96.8) among CHWs, and 97.8% (95% CI 93.3–100) among nurses.

The main recommendations outlined by the mothers/caretakers to ensure the success of the strategy were as follows: reinforce community outreach regarding the pre-referral treatment (46.7%), ensure the regular provision of artesunate suppositories (21.1%), reinforce the training of CHWs (14%) and offer pre-referral RA free or at low cost (8.4%).

### Adherence of the mothers/caretakers of CU5 to referral advice

The median age of the children enrolled in the study at the pre- and intervention periods was 2 years (IQR = 2) and 3 years (IQR = 2), respectively; for mothers/caretakers, it was 28 years (IQR = 13) and 31 years (IQR = 13), respectively. The adherence to referral advice at pre- and post-intervention was 94.1% (95% CI 91.7–96.4) and 84.3% (95% CI 80.6–88.1), respectively. Hence, adherence at pre-intervention was noninferior compared to adherence at post-intervention (Z = 4.87; p < 0.05).

Bivariate analysis showed that there were significant differences in adherence status according to the mothers’/caretakers’ education and marital status (p < 0.001) and whether the CU5 presented symptoms upon arrival at the CCS (p < 0.001) (Table 3).

**Table 3:** Respondent’s characteristics and symptoms of malaria shown by children under 5 years according to adherence status

	All respondents	Adhered to referral advice		Did not adhere to referral advice		p-value
		n	%	n	%	
Sex						
Female	294	247	84.0	47	16.0	0.913
Male	52	44	84.6	8	15.4	
Total	346	291	84.1	55	15.9	
Relationship to CU5						
Mother	279	237	84.9	42	15.1	0.382
Caretaker (father, sibling, grandmother, aunt, stepmother)	67	54	80.6	13	19.4	
Total	346	291	84.1	55	15.9	
Marital status						
Unmarried	18	9	50.0	9	50.0	0.001
Married	326	282	86.0	46	14.0	
Total	346	291	84.1	55	15.9	
Education						
No education	110	82	74.5	28	25.5	0.001
Educated	236	209	88.6	27	11.4	
Total	346	291	84.1	55	15.9	
Child’s symptoms on arrival at CCS						
Altered consciousness/convulsions	206	180	87.4	26	12.6	0.043
Vomiting everything/unable to eat, drink or suck/lethargic	140	111	79.3	29	20.7	
Total	346	291		55		

Multiple logistic regression analysis showed that the odds of adhering to referral advice were two times greater for educated mothers/caretakers than for mothers/caretakers with no education (OR 2.34, 95% CI 1.17–4.69).

**Fig. 1:**
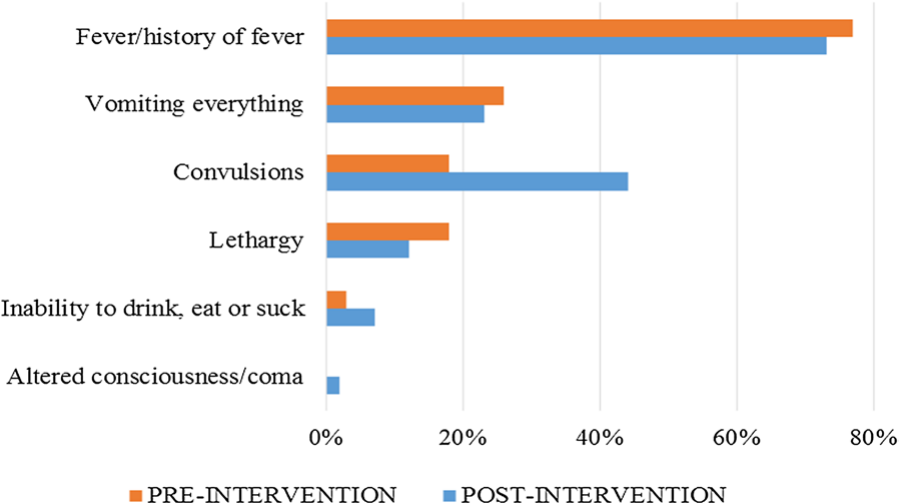
Symptoms and danger signs of malaria presented by the children when consulting at the community care site at pre-intervention (n = 386) and post-intervention (n = 346)

**Fig. 2:**
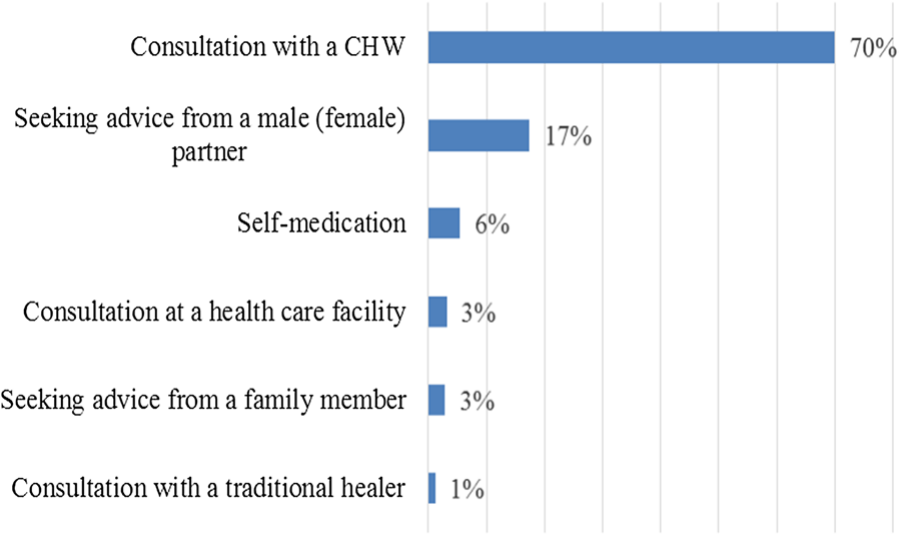
First care-seeking behaviours of mothers/caretakers of children under five following onset of danger signs of malaria at post-intervention (n = 346)

In addition, at post-intervention, the study examined some characteristics that underlie adherence to referral advice, including the following factors: symptoms presented by the children under five that led mothers/caretakers to consult a CHW; mothers’/caretakers’ care-seeking behaviours when a danger sign of malaria was presented by children under five; person who made the decision to consult a CHW or visit a health care facility; CHWs’ capacity to recognize danger signs of malaria in children under five and to give pre-referral treatment and referral advice. Figure 1 summarizes the presenting symptoms of CU5 upon consulting at the CCS. Figure 2 presents the first care-seeking behaviours following the onset of symptoms/danger signs of malaria at post-intervention. The majority of the respondents (70%) reported that they directly consulted a CHW, and the decision to do so was made mainly by the fathers (48%) or the mothers (48%). A total of 61.9% of mothers/caretakers consulted within 24 h after the onset of symptoms. Most of the mothers/caretakers who adhered to the referral advice (82.8%) reported that they arrived at the nearest health facility within 24 h after the RA was given to the child.

**Fig. 3:**
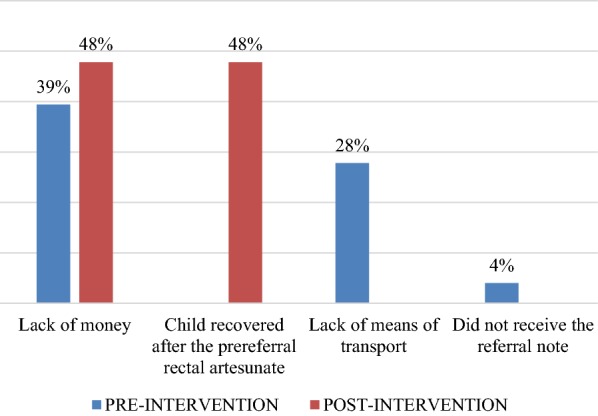
Reasons for non-adherence to referral advice at pre-intervention (n = 23) and post-intervention (n = 54)

Regarding the knowledge of CHWs for identifying cases with danger signs of malaria that were tested through scenarios involving hypothetical cases of CU5 presenting with various symptoms, 41.5% of the CHWs (n = 41) could correctly identify the five danger signs of malaria at post-intervention, compared to none at pre-test (p < 0.05). The reasons for not adhering to referral advice were diverse and are shown in Fig. 3 below:

## Discussion

The study findings confirm that the integration of RA for pre-referral treatment of severe malaria into the routine IMCI strategy was feasible and acceptable; acceptability was 79.4% among mothers, 90.3% among CHWs, and 97.8% among nurses. The high acceptability rate of this innovative intervention by the community is very important from a public health point of view, as the National Malaria Control Programme is expecting to deploy the RA for pre-referral treatment of severe malaria on a large scale.

The acceptability rate is similar to those observed in other studies [6–9]. This may be explained by the fact that although pre-referral RA was new in the selected communities, the use of a rectal route for the treatment of diseases was usual in these settings. This mode of treatment was also reported in other studies [7, 8]. The communities considered treatment via the rectal route to be more effective and easy to administer. These findings corroborate evidence documented elsewhere [8]. In contrast, in Laos, the use of rectal treatments was uncommon and was not considered to be more effective than treatment via other routes, even among health care workers [10].

Another explanation for the high acceptance of RA was that malaria was perceived by the community as a public health emergency and was considered to be responsible for most deaths among children. Moreover, confidence among the community in the health services provided by CHWs was observed.

Results showed that adherence to referral advice after integration of the pre-referral rectal artesunate was not more than 15% inferior compared to the initial adherence. This result strongly advocates recommendations to scale up the strategy throughout the country, as the findings stressed that introduction of the pre-referral rectal artesunate, which is a life-saving strategy, did not negatively affect adherence to referrals. In fact, failure to adhere to referral advice might delay identification of the real cause of the disease, while rapid recovery may lead to caretakers to perceive that no further treatment is necessary [8]. The high adherence to referral advice in targeted communities may be explained by the fact that these communities had longstanding PMI-funded malaria interventions, including community outreach aimed at raising community awareness of the importance of prompt care-seeking behaviours and promoting adherence to referral advice.

At the time the study was conducted, information on adherence to referral advice after receiving RA for the pre-referral treatment of severe malaria at the community level or on factors influencing adherence after pre-referral treatment was limited. Most of studies have assessed adherence to referrals from primary health facilities to hospitals, and it ranged from 28 to 75% [15–19].

Another study in Afghanistan assessed compliance with referrals for children with serious illness, including acute respiratory infections (ARI), diarrhoea or fever, as provided by any health workers at the community or at the health facility level (primary or secondary) and found that 76% of caregivers complied with the referral advice [14].

One factor that significantly influenced adherence to referral advice was the level of education of the mother/caretakers. Al Fadil found similar results in Sudan [16]. In addition, although the caregiver’s marital status and the child’s symptoms on arrival at CCS seemed to be associated with adherence to referral, the difference was not significant. These findings do not corroborate those of other studies [9, 11, 14–19]. In addition, for a referral to be completed, the parents of a sick child should seek treatment at the CCS or at health facility. This seems not to be an issue in the targeted health zones, as most respondents (more than 7 in 10) stated that they initially consulted a CHW or a health care provider when they noticed that their child presented a danger sign of malaria. However, as some of them acknowledged that they performed an inappropriate action, it is crucial to reinforce health education to improve care-seeking behaviour for sick children.

Considering that one of the key challenges in delivering health care services in the DRC is geographical accessibility, especially in remote rural areas where some villages are located more than 5 km (almost more than 1-h walking) from the nearest health care facility, the CHWs trained in IMCI offer a good alternative that extends primary health care to communities by maintaining linkages with formal health services.

CHWs are nonmedical volunteers who live in the catchment area they serve and can provide early treatment for diarrhea, pneumonia and malaria at any time of the day. However, this advantage is not always the case in other sub-Saharan African settings, as reported earlier in Malawi, where CHWs are government employees and live outside the catchment area [20]. Overall, in the DRC, the work of CHWs is well appreciated in the selected communities, and interventions such as pre-referral RA that are aimed at reinforcing the effectiveness of their job by decreasing the high mortality rate due to malaria in CU5 are likely to be accepted by both the community and the CHWs themselves.

However, failure for them to recognize danger signs of malaria as per the standardized IMCI case-definition would prevent expected health outcomes of the pre-referral RA strategy. That is, why Newbrander emphasized achieving successful child survival through recognition by health care providers of general danger signs of diseases that need referral [14]. The results showed that less than half CHW had the capacity to correctly identify the five danger signs suggestive of severe malaria as per the standardized IMCI case—definitions appropriate for the community level. With such a low proportion of CHWs who have the capacity to correctly identify all five danger signs of malaria, they may have a tendency to overrefer [14], or in a preventive point of view, they may overuse the pre-referral RA leading to drug resistance.

The risk of misclassification of suspected cases of severe malaria was more pronounced, with the results showing that one reason mothers/caretakers did not adhere to the referral advice is that the child recovered after the RA, which is extremely unlikely if the child had severe malaria and received just one single dose of RA. Finally, although the success of this strategy depends greatly on the adherence of mothers/caretakers to referral advice, the current findings also highlight the need to reinforce CHWs’ capabilities to distinguish symptoms suggestive of severe malaria from all other serious conditions that a CU5 may present to ensure prompt pre-referral treatment and to prevent overuse of the pre-referral RA [9].

## Limitations of the study

Adherence was assessed based on the self-reporting of the mothers/caretakers. Although the effective arrival time of the CU5 at the referral health facility was not checked, the CHWs systematically conducted follow-up interviews with the mothers/caretakers of all children who were given RA and referred. These visits enabled assessment of the child’s status and the collection of self-reported information regarding the referral facilities to which the child was taken. Other studies should more thoroughly explore adherence rates before and after the administration of pre-referral treatment at the community level, for instance, by providing a referral note that will be stamped at the facility on arrival and returned to the mothers/caretakers or by reviewing hospitalization registries at the health facility level.

## Conclusions

Integration of the pre-referral strategy for severe malaria into CCSs in the DRC is feasible and acceptable, and it positively affects adherence to referral advice. However, more health education is needed for parents of CU5 to raise awareness of the danger signs of malaria and reinforce prompt care-seeking behaviours. In addition, regular provision of RA and more training is needed for CHWs and nurses to increase their performance in the management of severe malaria at the community level.

## Data Availability

The datasets used and/or analysed during the current study are available from the corresponding author on reasonable request.

## Abbreviations

ACT: artemisinin-based combination therapy
BCZS: *Bureaux Centraux des Zones de Santé* (Health Zone Central Offices)
CCS: community care site
CHW: community health worker
CU5: children under 5 years old
DRC: Democratic Republic of the Congo
HZ: health zone
IEC: information, education and communication
IHP: integrated health project
IMCI: integrated management of childhood illnesses
IV: intravenous
MCZ: *Médecin Chef de Zone* (Chief Health Zone Doctor)
PHC: primary health care
PMI: President’s Malaria Initiative
NMCP: National Malaria Control Programme
RA: rectal artesunate
RDT: rapid diagnostic test
USAID: United States Agency for International Development
WHO: World Health Organization
